# Fabrication of 3D Porous and Flexible Thermoplastic Polyurethane/Carbon Nanotube Composites Towards High-Performance Microwave Absorption

**DOI:** 10.3390/molecules30173610

**Published:** 2025-09-03

**Authors:** Yanfang Li, Yandong Xu, Guangming Wen, Junwei Wang

**Affiliations:** 1 Department of Chemical and Material Engineering, Lyuliang University, Lyuliang 033001, China; xyandong2025@163.com (Y.X.); wgm@sxu.edu.cn (G.W.); 2 State Key Laboratory of Coal Conversion, Institute of Coal Chemistry, Chinese Academy of Sciences, Taiyuan 030001, China

**Keywords:** vapor-induced phase separation, thermoplastic polyurethane, carbon nanotube, microwave absorption

## Abstract

Materials with the characteristics of lightweight, thinness, flexibility, strong absorption, and broad bandwidth are of great concern in the microwave absorption field. Herein, a novel and facile technique, the vapor-induced phase separation (VIPS) method, was adopted to fabricate flexible thermoplastic polyurethane (TPU)/carbon nanotube (CNT) composites with a three-dimensional (3D) porous structure. The microstructure and electromagnetic wave absorption properties of the composites were tuned by varying the CNT weight ratio. The results show that the CNT established strong interfacial bonding with the TPU matrix. Different CNT weight ratios had a significant effect on the microstructure and electromagnetic parameters of the composites. The TPU/CNT composites achieved the minimum reflection loss (RL_min_) of −25.33 dB at 2.35 mm and an effective absorption bandwidth (EAB) of 4.89 GHz at 1.6 mm with a relatively low CNT weight ratio of 1 wt%. The conductive loss, dielectric loss, and multiple scattering synergistically contribute to favorable microwave absorption performances. This study showcases the use of a facile fabrication approach for the generation of flexible and porous TPU-based or other polymer counterparts-based functional composites via the VIPS method; it also paves the way for the large-scale application of high-performance microwave absorption materials.

## 1. Introduction

The rapid progress made in radar and electronic communication technologies in both military and civil contexts, such as stealth planes, missiles, generators, and sophisticated electronics, etc., has brought about paramount importance. However, the concomitant electromagnetic interference and pollution become a serious problem as they have a detrimental impact on the stable operation of electronic facilities and devices as well as human health. Microwave absorbing materials (MAMs) have thus emerged as an imperative strategy for their ability to convert electromagnetic wave energy to heat or other forms of energy. An ideal MAMs should possess the characteristics of “lightweight, thinness, bandwidth, and strong absorption” [1,2].

Based on this, researchers have been dedicated to high-performance MAMs. Carbon-based materials, such as carbon nanotubes (CNTs) [3], graphene [4], graphite [5], carbon fibers [6], MXene [7], and biomass carbon [8], have been extensively investigated due to their advantages of being lightweight, having favorable dielectric properties, and possessing excellent electron mobility. In order to fabricate MAMs that meet practical application requirements, carbon-based absorbers are usually composited with polymers, such as polyaniline (PANI) [9], polyimide (PI) [10,11,12], epoxy [13], polyurethane (PU) [14], or other types of polymers [15,16,17,18]. PU has received significant research attention, partly attributed to its diverse varieties including waterborne polyurethane (WPU) [19,20,21,22,23], polyurethane foam (PUF) [24,25], and thermoplastic polyurethane (TPU) [26], coupled with its advantages of oil resistance, high hardness, favorable mechanical strength, and substantial parametric freedom in compound blending and shaping methods. For example, Yan et al. [27] used the melting compounding method to create a CB/PLA-TPU composite bionic bamboo joint structure. This composite exhibited a minimum reflection loss (RL_min_) of −60.24 dB at a thickness of 2.58 mm and an effective absorption bandwidth (EAB) of 3.84 GHz. Zhang et al. [28] fabricated a CB/Ni/PLA/TPU composite by employing Fused Deposition Modeling (FDM) technology. The absorbers were carbon black (CB) and nickel (Ni), while the matrix was made of polylactic acid (PLA) and TPU. When the CB content was 5 wt%, the composite exhibited an RL_min_ of −34.14 dB (2 mm) and an EAB of 5.92 GHz. Solution blending is the most commonly used strategy for fabricating TPU-based composites. Sun et al. [29] prepared TPU-based composites via solution blending using MXene, CNTs, and Fe_3_O_4_ as the resources. The composite reached an RL_min_ of −54.81 dB and worked effectively from −20 to 100 °C. Ali et al. [30] utilized the solution blending method to synthesize multilayered composites using PU and polypropylene (PP) as the matrixes. A 4-layered polymer/graphene/epoxy composite achieved an RL_min_ of −20.02 dB. All these composites exhibit the advantages of flexibility. In addition, self-healing, anticorrosion, and sensing properties were also explored to optimize cross-domain adaptability [31,32,33,34].

Structural design, such as layered or porous structures, is an effective approach used to enhance the microwave absorption (MA) performances. Menon et al. [35] prepared porous PU membranes via the phase inversion technique using polyethylene glycol (PEG) as the pore-forming agent. The membranes showed a high shielding effectiveness of −32 dB at a thickness of 400 μm. Gao et al. [36] fabricated TPU/graphene flexible foam using the water-induced phase separation (WIPS) process. The composite showed an RL_min_ of −32.0 dB and an EAB of 4.7 GHz. Zheng et al. [37] employed room-temperature crosslinking to take advantage of PU’s self-foaming property. Among these pore-forming methods, the vapor-induced phase separation (VIPS) method, a technique where phase separation occurs in polymer solutions through non-solvent vapor, has attracted increased concern due to its advantages of having mild processing conditions (ambient temperature and atmospheric pressure) and a controllable microstructure. Compared to traditional phase separation methods, VIPS minimizes the use of organic solvent by leveraging vapor-induced precipitation, aligning with the green chemistry principles. VIPS has been adopted to fabricate composites applied in sensors [38] and for oil–water separation [39], etc. Our previous work prepared a series of TPU/graphene/Fe_3_O_4_ porous composites using the vapor-induced phase separation (VIPS) method. When the feed ratio of graphene is 3 wt %, TPU/G-3 composites exhibited an RL_min_ of −51.86 dB and an EAB of 4.28 GHz [40].

In this study, we innovatively combined TPU and CNT to fabricate 3D porous MAMs using the VIPS method. The process is remarkably facile under mild conditions. The solvent could be recycled, making it environmentally friendly. The interactions between TPU and the CNT were systematically analyzed via −C=O peak fitting from FT-IR. The pore size was measured via SEM. TG measurements were taken to evaluate the thermal stability. The impact of electromagnetic parameters, microstructure, and impedance matching on the MA performances was comprehensively investigated. Profiting from hierarchical pores derived from the VIPS process, the composite obtained the best MA performance (RL_min_: −25.33 dB, EAB: 4.89 GHz) at a relatively low CNT content of 1 wt%.

## 2. Results and Discussion

### 2.1. Morphology and Structure

**Figure 1:**
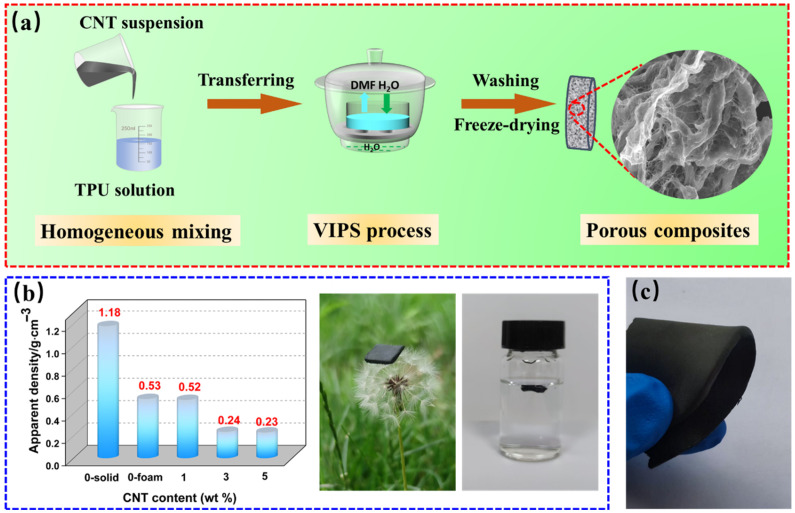
Schematic illustration for the fabrication process of TPU/CNT porous composites (**a**), the density and optical images of ultralight characteristics (**b**), and flexibility (**c**).

Figure 1a shows the preparation process for TPU/CNT porous composites. Herein, a facile VIPS method was introduced to fabricate the TPU/CNT 3D porous architecture. The microscale oxygen-containing groups from the CNT and the surfactant from the CNT slurry helped to evenly distribute the CNT throughout the TPU matrix. In the VIPS process, the non-solvent component, water, would condense on the surface of the TPU-based mixture and permeate into the polymer solution with the rapid evaporation of DMF, generating abundant and hierarchically gradient pores after freeze-drying. Figure 1b illustrates the ultralight properties of the composite film. A block of the sample could float on the surface of water and stand on the top of a dandelion. In particular, when CNT doping increases from 0 to 5 wt%, the density drops from 0.53 g·cm^−3^ to 0.23 g·cm^−3^. The composite films exhibit excellent flexibility, as they can be bent and recovered instantly, as shown in Figure 1c.

**Figure 2:**
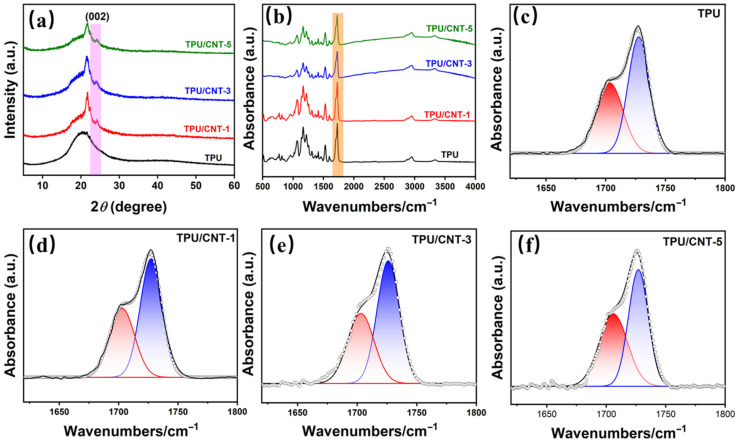
XRD patterns (**a**), FT-IR spectrum (**b**), and curves fitting of C=O stretching ((**c**–**f**) red: hydrogen-bonded carbonyl groups; blue: free carbonyl groups) of the samples.

XRD analysis elucidates the critical structure of the TPU-based composites, as shown in Figure 2a. The TPU matrix exhibits a broad peak at approximately 20.6°, which is the symbol of short-range order structure [41]. All the TPU/CNT composites reserved this peak, suggesting that the short-range structure was not altered by the filler incorporation. Additionally, the TPU/CNT composites show a peak at 24.3° (002), indicating that the CNT was successfully incorporated into the composites [42]. The XRD results indicate the successful preparation of TPU/CNT composites.

FT-IR analysis was carried out to better clarify the changes in the interaction with the incorporation of the CNT, as shown in Figure 2b. All of the samples had a prominent peak at 1725 cm^−1^, which is attributed to the −C=O stretching vibration peak of polyol and carbamate. Notably, the peak shape shows a distinct change with the increase in CNT content, suggestive of non-negligible hydrogen bonding effects. To better clarify the effect of hydrogen bonding, peak fittings were conducted in the range of 1600~1800 cm^−1^, as shown in Figure 2c–f and Table S1. The −C=O peak is fitted into two peaks, including a hydrogen-bonded carbonyl group (~1703 cm^−1^) and a free carbonyl group (~1726 cm^−1^). The hydrogen bonding degree is quantized as the hydrogen bonding index (HBI), which can be calculated with the peak area ratio of the hydrogen-bonded carbonyl group and the free carbonyl group. As shown in Table S1, the HBI of TPU, TPU/CNT-1, TPU/CNT-3, and TPU/CNT-5 is 0.652, 0.675, 0.737, and 0.896, respectively, exhibiting an escalating tendency as the CNT content increases. This indicates that the residual groups from the CNT slurry establish an intense interaction with TPU, endowing the composites with a homogeneous structure [43].

**Figure 3:**
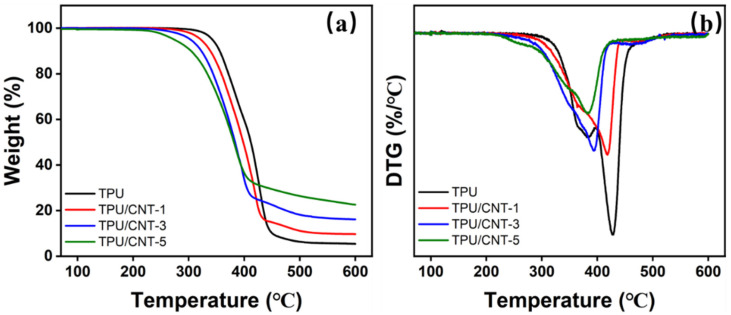
TG (**a**) and DTG (**b**) curves of TPU and TPU/CNT composites.

**Figure 4:**
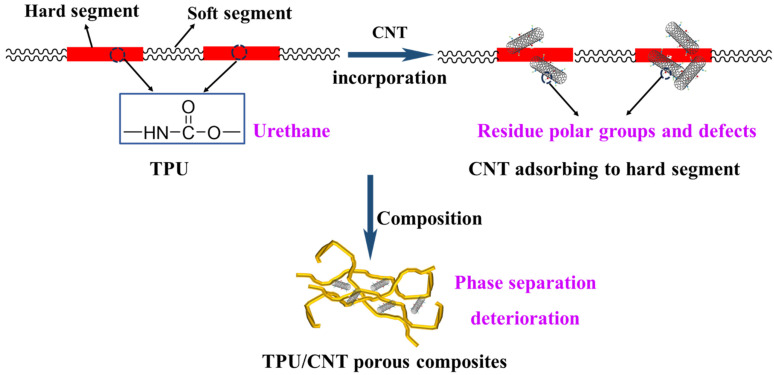
Schematic illustration of the influence of CNT introduction on the phase structure of TPU.

Thermal stability is a significant parameter for the assessment of materials in practical applications. Figure 3 and Table S2 showed the results of the TG and DTG analyses of TPU and TPU/CNT composites. The addition of CNT results in a notable and significant difference. As a result of the interaction between the CNT and TPU, the initial degradation temperature (5% weight loss, T_5%_) for TPU, TPU/CNT-1, TPU/CNT-3, and TPU/CNT-5 drops from 349.8 °C, 330.6 °C, and 312.8 °C to 288.1 °C, respectively. T_10%_ and T_50%_ exhibit the same tendency, as shown in Table S2. The composites show a declining trend as CNT doping increases, mostly because of their interaction with TPU, even if the CNT has the potential to boost thermal stability. The hard segment of TPU is primarily responsible for its thermal stability. This enhancement of thermal stability contributed as a result of CNT incorporation is inferior to the influence of structural changes on hard segments. As seen in Figure 3b, the remaining polar groups and defects from the CNT slurry specifically cause interaction between the CNT and the hard segment of TPU, deteriorating the microphase separation and resulting in worse thermal stability. TPU exhibits two peaks, noted as the maximum decomposition temperature (T_max_), which appear at 384.6 °C and 428.8 °C corresponding to the degradation of the soft and hard segments, respectively. However, all the TPU/CNT composites show only one T_max_ peak appeared at 418.3 °C, 393.7 °C and 382.4 °C for TPU/CNT-1, TPU/CNT-3 and TPU/CNT-5, respectively, indicating the deterioration of microphase separation, which is consistent with the tendency of decreasing thermal stability with an elevated CNT mass ratio. In addition, the residue exhibits a distinct differences of 5.54%, 9.99%, 16.09, and 22.47% for TPU, TPU/CNT-1, TPU/CNT-3, and TPU/CNT-5, which is positively correlated to the incorporation of CNT [30]. The relevant mechanism of the TG results is shown in Figure 4.

**Figure 5:**
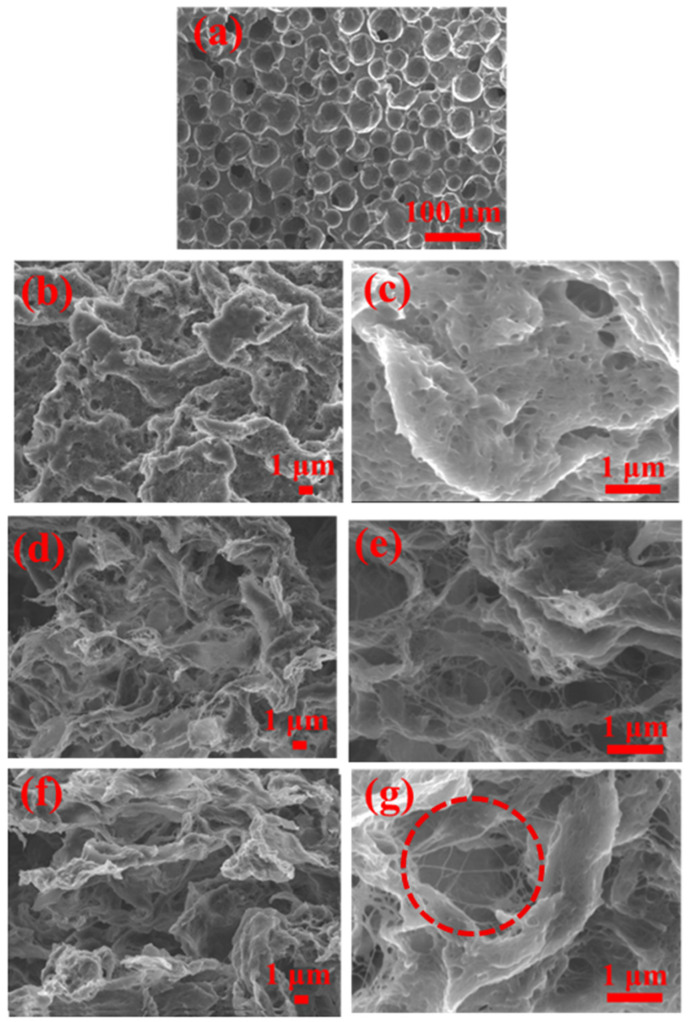
Cross-section SEM micrographs of TPU (**a**), TPU/CNT-1 (**b**,**c**), TPU/CNT-3 (**d**,**e**), and TPU/CNT-5 (**f**,**g**).

Figure 5 illustrates the microstructure of the samples. All the samples exhibit a hierarchically interconnected three-dimensional open-cell structure. It is evident that the introduction of CNT plays a significant role in the formation of pores [38,39]. Specifically, TPU foam exhibits uniform and regular pores with a mean pore size of 39.69 μm. Whereas the mean pore size shows sharp decreases to 0.48 μm, 0.55 μm, and 1.20 μm for TPU/CNT-1, TPU/CNT-3 and TPU/CNT-5, respectively, suggestive of the effect that nucleation had on the CNT in the pore formation process. Based on Henry’s law, higher solution concentrations means lower DMF vapor pressure, resulting in a lower evaporation rate and then a higher surface temperature, lowering the temperature differential between the atmosphere and surface (ΔT). The relationship of ΔT, water droplet radius (R), and the growth time (t) is as follows: $\mathrm{dR}/\mathrm{dt}=\propto \Delta T^{0.8}$. According to this relationship, the incorporation of the CNT endows the mixture with a higher concentration and thus a much lower pore size, but this tendency becomes abnormal when the CNT concentration surpasses 3 wt%. This is attributed to the fact that the pores are not only inherited from the VIPS process but also mutual entanglements among CNT and TPU chains, which could be clearly seen in Figure 5g. These entanglements generated relatively larger pores compared with those generated with the VIPS process, as shown in the red dashed border in Figure 5g, resulting in an increase in the mean pore size and a more uneven distribution [44].

### 2.2. Microwave Absorption Performance

**Figure 6:**
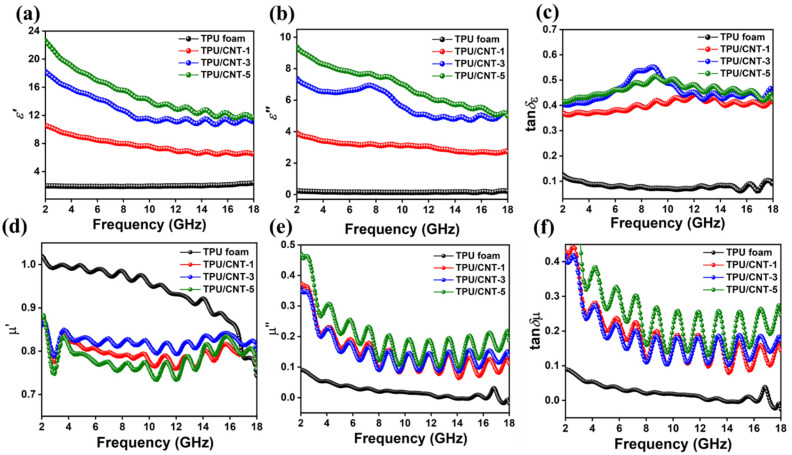
Frequency dependences of real and imaginary parts of permittivity (**a**,**b**), real and imaginary parts of permeability (**d**,**e**), dielectric loss tangents (**c**), and magnetic loss tangents (**f**) of TPU and TPU/CNT composites in 2–18 GHz.

The frequency-dependent electromagnetic parameters were comprehensively analyzed, as shown in Figure 6. The electromagnetic parameters include complex permittivity (ε_r_ = ε′ − jε″) and complex permeability (μ_r_ = μ′ − jμ″). The real parts (ε′ and μ′) correlate to the electric and magnetic energy storage capabilities, and the imaginary parts (ε″ and μ″) represent the dissipation of electric and magnetic energy, respectively. The dielectric loss mechanism between ε′ and ε″ can be elucidated according to the Debye theory, as shown in Equations (1) and (2):

(1) $$\varepsilon^{\prime }=\varepsilon_{\infty }+\frac{\varepsilon_{s}-\varepsilon_{\infty }}{1+w^{2}\tau^{2}}$$

(2) $$\varepsilon^{\prime \prime }=\frac{\varepsilon_{s}-\varepsilon_{\infty }}{1+w^{2}\tau^{2}}w\tau +\frac{\sigma }{w\varepsilon_{0}}$$

where τ represents the relaxation time, w is the angular frequency, σ is the conductivity, and $\varepsilon_{s}$, $\varepsilon_{\infty }$, and $\varepsilon_{0}$ refer to the static permittivity, relative permittivity at the high-frequency limit, and dielectric constant in vacuum, respectively. As shown in Figure 6a, the ε′ and ε″ of TPU are the lowest at approximately 1.9 and 0.16, respectively, demonstrating insignificant electromagnetic wave energy storage and dissipation capacities. The ε′ value exhibits an upward trend with increased CNT content, rising from 10.58 to 6.57 for TPU/CNT-1, 18.22 to 11.32 for TPU/CNT-3, and 22.59 to 11.78 for TPU/CNT-5, respectively. Incorporating conductive CNTs endows the composites with free electrons, heterointerfaces, and dipoles. The conductive network gradually developed as the CNT content increased. These unbound free electrons would polarize under electromagnetic fields, enhancing dielectric loss. Meanwhile, interfacial polarization and dipolar polarization were also triggered to store energy. The abundant pores also play a significant role in the variation in ε′. The hierarchical pores becomes richer with elevated CNTs. These air-filled pores would lead to the reduction in ε′. However, higher conductivity, as well as enhanced interfacial polarization at pore–CNT boundaries, could partially offset this reduction. The combined effects exhibit an upward tendency with elevated CNT content. Furthermore, the ε′ of all TPU/CNT composites exhibits a reduction as the frequency rises, suggestive of typical frequency-dependent dielectric characteristics. This is ascribed to the difficulties encountered in the frictional rotation of dipoles. These dipoles fail to synchronize with the alternating electromagnetic field, exhibiting a phase lag and thereby frequency dispersion effect. The ε″ shows an analogous upward trend with ε′ as CNT increases, rising from 3.97 to 2.72 for TPU/CNT-1, 7.37 to 5.00 for TPU/CNT-3, and 9.36 to 5.00 for TPU/CNT-5, respectively.

According to the free electron theory, higher CNT content endows the composite with more conductive paths for electron migrating and transferring, contributing to stronger conductive loss. Except for conductive loss, enhanced interfacial and dipole polarization relaxation also contributes to overall dielectric loss. In terms of the magnetic properties, because there are no magnetic components in any of the composites, μ′ and μ″ are approximately 1 and 0 [45].

**Figure 7:**
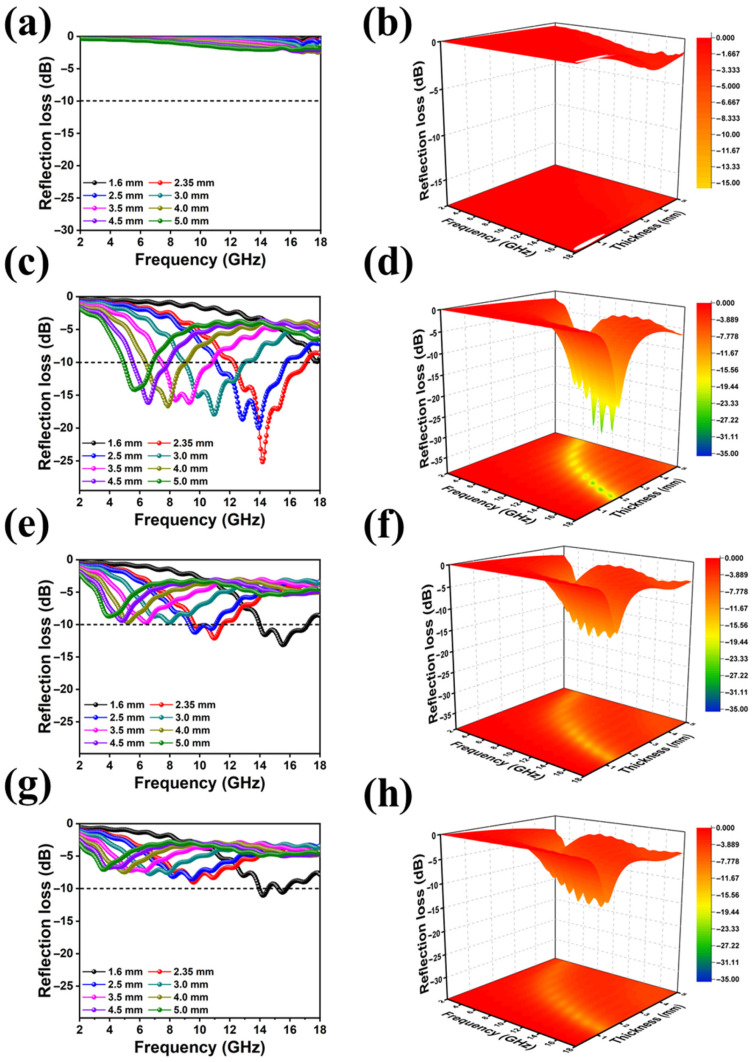
RL curves and corresponding 3D plots of TPU (**a**,**b**), TPU/CNT-1 (**c**,**d**), TPU/CNT-3 (**e**,**f**), and TPU/CNT-5 (**g**,**h**).

The dielectric loss tangent (tanδε = ε″/ε′) and magnetic loss tangent (tanδµ = μ″/μ′) were investigated to further quantify the contribution of dielectric and magnetic loss, as shown in Figure 7c,f. In Figure 7c,f, the tanδε values of all the TPU/CNT composites are above 0.35, while the average tanδµ value is only 0.2 due to the absence of magnetic components. tanδε is much higher than tanδµ, indicating that dielectric loss is the dominant dissipation mechanism.

To investigate the impact of the CNT content on the MA performances of the composites, the frequency-dependent RL curves and corresponding 3D plots in 2–18 GHz were calculated according to the transmission line theory, as expressed in Equations (3) and (4):

(3) $$Z_{in}=Z_{0}{\left(\mu_{r}/\varepsilon_{r}\right)}^{0.5}\tanh\left(2\pi jfd/c\sqrt{\mu_{r}\varepsilon_{r}}\right)$$

(4) $$RL(dB)=20\log\left|\frac{Z_{in}-Z_{0}}{Z_{in}+Z_{0}}\right|$$

where $Z_{0}$ represents the characteristic impedance of free space, $Z_{\mathrm{in}}$ is the input impedance, $\varepsilon_{r}$ and $\mu_{r}$ denote the complex permittivity and complex permeability, d is the thickness of the absorbers, f represents the microwave frequency, and c is the velocity of light in free space, respectively. Except for RL, EAB is another significant parameter for the assessment of MA performance. EAB represents the frequency range covered by RL lower than −10 dB, which represents 90% microwave absorption [46]. Figure 7 displays the RL curves and corresponding 3D plots. As shown in Figure 7a,b, TPU foam demonstrates almost no electromagnetic absorption, suggesting that TPU foam is an electromagnetically transparent material. CNT incorporation endows the composites with a dramatic improvement in the MA properties. In particular, TPU/CNT-1 achieved the best MA performance among all the TPU/CNT composites with the RL_min_ of −25.28 dB in 2.35 mm and the EAB of 4.89 GHz (12.23 GHz–17.12 GHz) in 2.5 mm due to balanced impedance matching and moderate dielectric loss, whereas, the composites demonstrate a nonlinear relationship between CNT content and MA performance. The MA performance of TPU/CNT performances drastically declined when the CNT content increased to 3 wt% and 5 wt%. Specifically, the RL_min_ value of TPU/CNT-3 composite only reached −13.18 dB, while the value declined to −11.09 dB for TPU/CNT-5. Although higher CNT content endows the composites with a stronger dissipation capability, as shown in Figure 6, this also leads to impedance mismatching. The results demonstrate that the electromagnetic parameters and microstructure have a significant impact on the MA performances of the samples.

The correlation of ε′ and ε″ was summarized to analyze the polarization relaxation loss mechanism based on the Debye relaxation theory, as shown in Equation (5):

(5) $${\left(\varepsilon^{\prime }-\frac{\varepsilon_{s}+\varepsilon_{\infty }}{2}\right)}^{2}+{\left(\varepsilon^{\prime \prime }\right)}^{2}={\left(\frac{\varepsilon_{s}-\varepsilon_{\infty }}{2}\right)}^{2}$$

**Figure 8:**
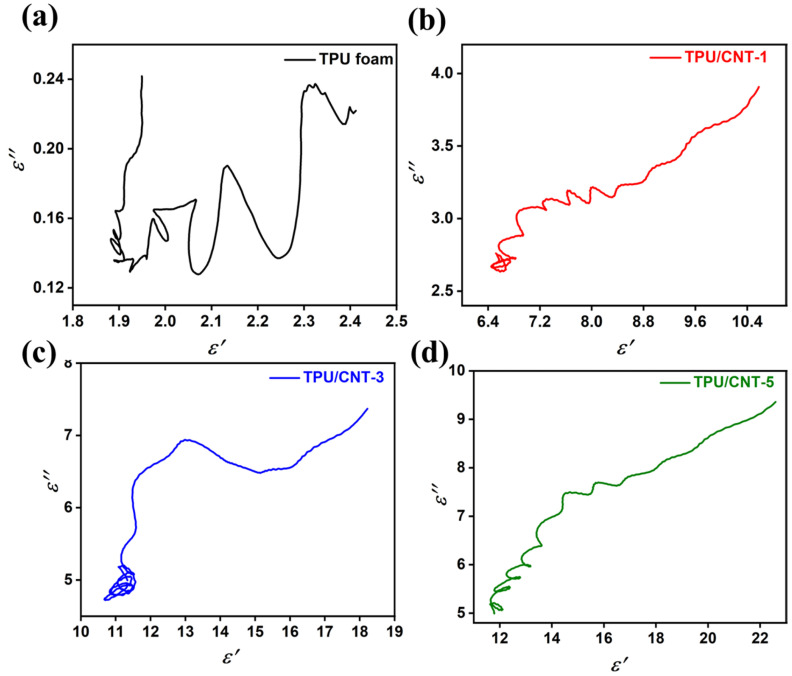
Cole–Cole curves of TPU (**a**) and TPU/CNT composites (**b**–**d**).

According to Equation (5), a semicircle signifies one dielectric relaxation process, known as a Cole–Cole semicircle. Meanwhile, the straight line at the end of the Cole–Cole curve indicates the existence of conductive loss [47]. Three different Cole–Cole semicircles in TPU are depicted in Figure 8a, indicating the presence of some polarization relaxation processes. Introducing the CNT resulted in a great difference in the ε″-ε′ curves. For instance, the semicircles became more noticeable as the CNT content rose, indicating that more dipoles and interfacial polarizations were involved. Also, the straight line became longer, indicating that conductive loss became stronger as the CNT content increased [26,40].

The attenuation constant (α) is the most intuitive and quantitative parameter for evaluating the dissipation performance, reflecting on the ability of an absorber to convert electromagnetic wave energy into other forms of energy. The α was calculated according to Equation (6):

(6) $$\alpha =\frac{\sqrt{2}\pi f}{c}\sqrt{\left(\mu^{\prime \prime }\varepsilon^{\prime \prime }-\mu^{\prime }\varepsilon^{\prime }\right)+\sqrt{{\left(\mu^{\prime \prime }\varepsilon^{\prime \prime }-\mu^{\prime }\varepsilon^{\prime }\right)}^{2}+{\left(\mu^{\prime }\varepsilon^{\prime \prime }-\mu^{\prime \prime }\varepsilon^{\prime }\right)}^{2}}}$$

**Figure 9:**
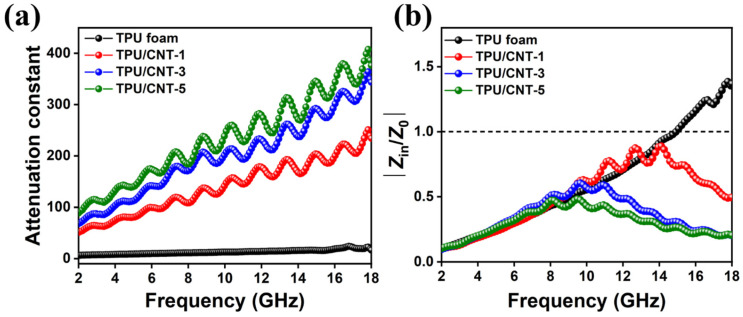
The attenuation constant (**a**) and impedance matching characteristics at 2.35 mm (**b**) of TPU and TPU/CNT composites.

As shown in Figure 9a, all the samples exhibit an upward trend with the increased CNT content. Specifically, the α value spans from 6.35 to 16.59, 51.05 to 234.20, 68.40 to 343.54, and 88.99 to 376.17 for TPU, TPU/CNT-1, TPU/CNT-3, and TPU/CNT-5, respectively. The results indicate that more CNT content would endow the composites with stronger capabilities for attenuating the electromagnetic waves, whereas the MA performance is not directly proportional to the variation in α since more CNT content would lead to more inferior impedance matching. Although multiple scattering and multiple interfaces polarization are enhanced due to more abundant pores with increased CNT content, the impedance mismatching induced by higher conductivity becomes the dominant factor for the enhancement of MA performance. Inferior impedance matching means that more incident electromagnetic waves would be reflected at the surface of an absorber rather than permeating into its interior. Therefore, assessing the impedance matching ability is of great significance except for the attenuation constant. The impedance matching of the samples at a thickness of 2.35 mm is illustrated in Figure 9b. In Figure 9b, TPU/CNT-1 achieved the best MA performance among all the samples. The favorable impedance matching and moderate attenuation constant collaboratively contribute to benign MA performances [48]. These results are consistent with the RL values shown in Figure 6.

In order to facilitate a more comprehensive and intuitive comparison of the MA performance among the representative CNT-based composites, loading ratio, RL_min_, and EAB, a series of CNT-based composites were exhibited, as shown in Table 1. In Table 1, the RL_min_ and EAB of TPU/CNT-1 are rather competitive (−25.33 dB, 4.89 GHz) among its counterparts. Notably, the CNT content is the lowest, demonstrating that the presence of pores originating from the VIPS process could significantly reduce the filler content required to achieve a comparable MA performance.

### 2.3. Microwave Absorption Mechanism

**Figure 10:**
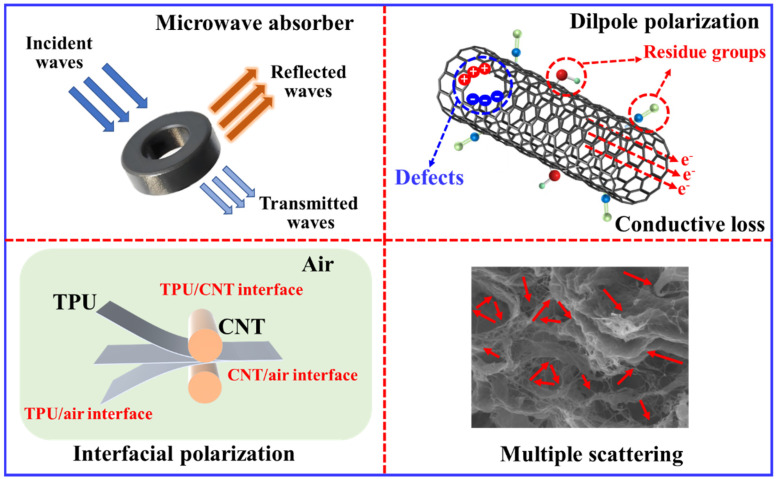
Schematic illustration of the microwave absorption mechanism of TPU/CNT porous composites.

Figure 10 illustrates the MA mechanism. First, conductive networks are introduced into the composite via a highly conductive CNT. These conductive networks make it possible for free electron migrating and hopping, contributing to the conductive loss. Second, the polar groups from TPU (such as –NHCOO– from the hard segment and –COO– from the soft segment) and the residue groups and defects from CNT slurry would incorporate abundant dipoles, which polarize, consuming energy through dipole polarization and relaxation loss. The simultaneous generation of several heterogeneous interfaces, including CNT/TPU interfaces, CNT/air interfaces, and TPU/air interfaces, contributes interface polarization loss to further dissipate electromagnetic waves. In addition, 3D multiple pores enable EM waves to scatter repeatedly inside the sample, attenuating EM wave energy to other forms of energy. In summary, the unique 3D porous structure endows the TPU/CNT composites with high-performance MA performances at a low CNT content via multiple attenuation mechanisms [54].

## 3. Materials and Methods

### 3.1. Materials

TPU pellets (75A) were purchased from Wanhua Chemical Group Co., Ltd. (Yantai, Shandong, China). CNT/DMF slurry (CNT content: 0.2 wt%, surfactant content: 0.31 wt%) was obtained from Jiacai technology Co., Ltd. (Chengdu, Sichuan, China). DMF was purchased from Sinopharm Chemical Reagent Co., Ltd. (Shanghai, China). Deionized water was obtained via a water purification system (EPED, GREEN-Q3-20T, Nanjing, Jiangsu, China). All chemicals and solvents were employed as obtained without any additional purification.

### 3.2. Preparation of 3D Porous TPU/CNT Composites

Three-dimensional porous TPU/CNT composites were fabricated using the VIPS approach. TPU was first dissolved in DMF to prepare a solution with a solid content of 20%. The samples with 1, 3, and 5 wt% CNT loadings were fabricated by incorporating 5.13 g, 16.24 g, and 28.65 g of CNT slurry into the 5 g of TPU solution, respectively. The solid content of the mixture (TPU, CNT, and surfactant) for the samples of 1 wt% and 3 wt% was adjusted to be the same as that of the 5 wt % sample by supplementing DMF. The mixture was sonicated for 30 min (120 W), followed by vigorous magnetic stirring for 12 h to guarantee homogeneous dispersion of CNT in the mixture. The mixture was then poured into a glass vessel and transferred to a sealed desiccator with the appropriate amount of deionized water (not passing through the partition layer) at the bottom to undergo the VIPS process. The VIPS process was performed at room temperature. The solid material was taken out until no further precipitation was observed after 7 days. This material was then washed repeatedly with deionized water for 5 times to remove the residual DMF, followed by lyophilization at the freeze-drying temperature of −40 °C and the pressure of 0.05 Pa for 48 h. The mass ratios of CNT 1 wt%, 3 wt%, and 5 wt% composites were marked as TPU/CNT-1, TPU/CNT-3, and TPU/CNT-5, respectively.

### 3.3. Characterization

The phase construction of the samples was analyzed using X-ray diffractometer (XRD, Bruker Advance D8, Bruker, Billerica, MA, USA) with Cu K radiation (40 kV, 40 mA, λ = 1.5406 Å). The chemical structures of the samples were analyzed using Fourier transform infrared spectrometer (FT-IR, Nicolet 380, Thermo Fisher Scientific, Waltham, MA, USA) in the attenuated total reflection (ATR) mode in the range of 4000~500 cm^−1^. The thermal properties of the samples were measured using a thermogravimetric analyzer (TGA, TGA-50, Shimadzu Scientific Instruments, Kyoto, Japan) under N_2_ atmosphere. The samples were heated to 100 °C and maintained for 30 min to ensure that water was completely removed, and then they were heated to 600 °C at a heating rate of 10 °C/min. The cross-sectional morphologies of the samples were observed using a field-emission scanning electron microscope (SEM, JSM-7900F, JEOL, Tokyo, Japan) with an accelerating voltage of 5 kV. The mean pore size was measured using Nano Measurer 1.2 based on SEM images. The density of the samples was measured using a pycnometer method [40].

The electromagnetic parameters, including the complex permittivity and complex permeability, were tested using a vector network analyzer (VNA, E5071C, Agilent Technologies, Santa Clara, CA, USA) according to the coaxial line method. The composite film was shaped into a coaxial ring with an outer diameter of 7 mm and an inner diameter of 3.04 mm.

## 4. Conclusions

This study demonstrates the successful fabrication of lightweight, porous TPU/CNT composites via the VIPS method, achieving tunable MA properties through controlled CNT incorporation. The CNT played a significant role in the pore size distribution and composite density (0.53–0.23 g·cm^−3^) and established strong interfacial bonding with the TPU matrix. The presence of pores endowed the composite with favorable MA performance at a relatively low CNT content of 1 wt%. TPU/CNT-1 exhibits the best MA performances with an RL_min_ of −25.33 dB at 2.35 mm and an EAB of 4.89 GHz at 1.6 mm. This study offers a facile, scalable, and solvent-efficient method to fabricate porous flexible polymer composites with potential applications in aerospace, wearable electronics, and stealth technologies. The strategic design of multicomponent dielectric–magnetic architectures with optimized synergy effects, coupled with dynamic environment-responsive performance tuning, constitutes a transformative research paradigm for developing ultra-broadband electromagnetic wave absorbers. This study advances the rational design of porous MA materials and establishes VIPS as a versatile platform for the production of next-generation functional composites.

## Figures and Tables

**Table 1:** Comparison of microwave absorption properties of representative CNT-based composites.

Absorber	Matrix	Loading/wt%	Thickness/mm	RL_min_/dB	EAB/GHz	Ref
CNT/iron	paraffin	15	2.92	−58.56	5.6	[10]
Co@C/CNTs	PVDF	15	3.01	−57.6	5.4	[3]
CNT	PU	0.1–0.5	2.3	−6	0	[49]
CNT	TPU	5	1.87	−41.66	2.73	[6]
TiO_2_/C/Co@C	paraffin	30	1.8	−41.35	5.06	[50]
CNT	PU	5	2	−22	8.7	[51]
CNT	PU	5	1	−36	~3	[52]
Ti@MWCNT/Fe	TPU	30	2	−42.53	4.2	[53]
CNT	TPU	1	2.35	−25.33	4.89 (1.6 mm)	This work

## Data Availability

Data are contained within the article and Supplementary Materials.

## Supplementary Materials

The following supporting information can be downloaded at https://www.mdpi.com/article/10.3390/molecules30173610/s1. Figure S1: The SEM (a) and TEM (b) micrographs of the CNT. Table S1: Hydrogen bonging index and relative areas of the carbonyl stretching region in FT-IR spectra. Table S2: Thermal characteristics of TPU and TPU-based composites from TG results.

## References

[1] Lv H.L., Yang Z.H., Pan H.G., Wu R.B. (2022). Electromagnetic absorption materials: Current progress and new frontiers. Prog. Mater. Sci..

[2] Zhao B., Yan Z.K., Liu L.L., Zhang Y.Y., Guan L., Guo X.Q., Li R.S., Che R.C., Zhang R. (2024). A liquid-metal-assisted competitive galvanic reaction strategy toward indium/oxide core−shell nanoparticles with enhanced microwave absorption. Adv. Funct. Mater..

[3] Wang Y.L., Zhao P.Y., Liang B.L., Chen K., Wang G.S. (2023). Carbon nanotubes decorated Co/C from ZIF-67/melamine as high efficient microwave absorbing material. Carbon.

[4] Liu Z., Wang B., Wei S.C., Huang W., Wang Y.J., Liang Y. (2024). Review of three-dimensional graphene and related composite materials for microwave absorption. ACS Appl. Nano Mater..

[5] Matzui L.Y., Syvolozhskyi O.A., Vovchenko L.L., Yakovenko O.S., Len T.A., Ischenko O.V., Vakaliuk A.V., Oliynyk V.V., Zagorodnii V.V., Naumenko A. (2024). Segregated conductive polymer composite with Fe_3_O_4_-decorated graphite nanoparticles for microwave shielding. Materials.

[6] Kasgoz A., Korkmaz M., Durmus A. (2019). Compositional and structural design of thermoplastic polyurethane/carbon based single and multi-layer composite sheets for high-performance X-band microwave absorbing applications. Polymer.

[7] Li R.S., Gao Q., Xing H.N., Su Y.Z., Zhang H.M., Zeng D., Fan B.B., Zhao B. (2021). Lightweight, multifunctional MXene/polymer composites with enhanced electromagnetic wave absorption and high-performance thermal conductivity. Carbon.

[8] Han X.J., Feng Y.F., Zhang N., Du W., Zhang W.L., Yu Q.Q., Liu Y.F., Wang B., Jiang F.Y., Liu L.Y. (2025). Bio-based multifunctional carbon aerogels for ultrawide electromagnetic wave absorption and thermal insulation. Carbon.

[9] Wei H.W., Dong J.D., Fang X.J., Zheng W.H., Sun Y.T., Qian Y., Jiang Z.X., Huang Y.D. (2019). Ti_3_C_2_T_x_ MXene/polyaniline (PANI) sandwich intercalation structure composites constructed for microwave absorption. Compos. Sci. Technol..

[10] Li S.S., Ma T.T., Zhu M.Y., Lu Y.Z., Tang X.W., Li W., Hong W., Yang S.Y., Li Y.F., Zheng P.L. (2025). Magnetic isocyanate-based polyimide composite foam for efficient microwave absorption. Compos. Part B Eng..

[11] Ting T.H., Jau Y.N., Yu R.P. (2012). Microwave absorbing properties of polyaniline/multi-walled carbon nanotube composites with various polyaniline contents. Appl. Surf. Sci..

[12] Zhou C.Q., Ni L., Lu J.Y., Meng H.C., Luo Y.F., Liang M., Zou H.W. (2024). Robust and multifunctional polyimide composite foams via microsphere foaming towards efficient thermal insulation and broadband microwave absorption. Polymer.

[13] Guo T., Chen X.L., Zeng G.J., Yang J.L., Huang X.Z., Li C.G., Tang X.Z. (2021). Impregnating epoxy into N-doped-CNTs@carbon aerogel to prepare high-performance microwave-absorbing composites with extra-low filler content. Compos. Part A Appl. Sci. Manuf..

[14] Zhu X.J., Dong Y.Y., Xiang Z., Cai L., Pan F., Zhang X., Shi Z., Lu W. (2021). Morphology-controllable synthesis of polyurethane-derived highly cross-linked 3D networks for multifunctional and efficient electromagnetic wave absorption. Carbon.

[15] Anjana, Chandra A. (2023). Facile synthesis and characterization of polymer composites with cobalt ferrite and biomass based activated carbon for microwave absorption. Mater. Today Commun..

[16] Deng Y.M., Ren B., Jia Y.J., Wang Q., Li H.J. (2024). Layered composites made of polymer derived SiOC/ZrB_2_ reinforced by ZrO_2_/SiO_2_ fibers with simultaneous microwave absorption and thermal insulation. J. Mater. Sci. Technol..

[17] Ling Q.C., Sun J.Z., Zhao Q., Zhou Q.Y. (2009). Microwave absorbing properties of linear low density polyethylene/ethylene–octene copolymer composites filled with short carbon fiber. Mater. Sci. Eng. B.

[18] Phadtare V.D., Parale V.G., Lee K.Y., Kim T., Puri V.R., Park H.H. (2019). Flexible and lightweight Fe_3_O_4_/polymer foam composites for microwave-absorption applications. J. Alloys Compd..

[19] Bi S., Song Y.Z., Hou G.L., Li H., Liu Z.H., Hou Z.L., Zhang J.Y. (2023). Sandwich nanoarchitectonics of heterogenous CB/CNTs honeycomb composite for impedance matching design and microwave absorption. J. Alloys Compd..

[20] Dai M.W., Zhai Y.H., Wu L., Zhang Y. (2019). Magnetic aligned Fe_3_O_4_-reduced graphene oxide/waterborne polyurethane composites with controllable structure for high microwave absorption capacity. Carbon.

[21] Duan Y.P., Liu Y., Cui Y.L., Ma G.J., Wang T.M. (2018). Graphene to tune microwave absorption frequencies and enhance absorption properties of carbonyl iron/polyurethane coating. Prog. Org. Coat..

[22] Sheng A., Ren W., Yang Y.Q., Yan D.X., Duan H.J., Zhao G.Z., Liu Y.Q., Li Z.M. (2020). Multilayer WPU conductive composites with controllable electro-magnetic gradient for absorption-dominated electromagnetic interference shielding. Compos. Part A Appl. Sci. Manuf..

[23] Xu Y.D., Lin Z.Q., Yang Y.Q., Duan H.J., Zhao G.Z., Liu Y.Q., Hu Y.G., Sun R., Wong C.P. (2022). Integration of efficient microwave absorption and shielding in a multistage composite foam with progressive conductivity modular design. Mater. Horiz..

[24] Zhang C.M., Li H., Zhuo Z.Z., Dugnani R., Sun C.Y., Chen Y.J., Liu H.Z. (2017). Facile fabrication of ultra-light and highly resilient PU/RGO foams for microwave absorption. RSC Adv..

[25] Zheng X.Y., Zhang H.W., Jiang R.J., Liu Z.H., Zhu S.S., Li W.Y., Jiang L., Zhou X. (2023). Lightweight polyurethane composite foam for electromagnetic interference shielding with high absorption characteristic. J. Colloid Interface Sci..

[26] Zhang W., Li K., Han L., Wu T., Zhang J., Cheng J. (2024). Ultra-strength polyurethane/MOF-derived composites with self-healing and recycling capabilities and highly efficient microwave absorption properties. J. Mater. Chem. C.

[27] Yan T.M., Ye X.C., He E.Y., Gao Q., Wang Y.M., Gong L., Ye Y.S., Wu H.H. (2024). Preparation of a double-layer bionic bamboo structure absorber based on CB/PLA-TPU composites and its broadband microwave absorption characteristics. J. Alloys Compd..

[28] Zhang F.X., Liu S.K., Chao B., Deng S.X., Zhou Y.M., Wu H.H., Wang Q.S. (2025). Study on FDM preparation and properties of carbon black/nickel/polylactic acid/thermoplastic polyurethane electromagnetic wave absorbing composites. Compos. Commun..

[29] Sun Z.X., Shen J.H., Chen W., Chen Y.M., Li X.P., Zheng J.J., Jiang S.H., Zhou L.J. (2024). High-performance polyurethane composite elastomers with coral reef-like hierarchical heterostructures for electromagnetic wave absorption and flexible sensing. Mater. Today Chem..

[30] Ali M.S., Santhosi B.V.S.R.N., Garugubilli R., Syed J. (2025). Effective microwave absorbing polymer nanocomposites in X-band: The role of graphene in polyurethane/polypropylene multilayered structures. Vacuum.

[31] Han Q.Q., Wang S., Cheng X., Du X.S., Wang H.B., Du Z.L. (2023). Self-healing polyurethane coating based on porous carbon/Ni hybrid composites for electromagnetic wave absorption. Compos. Part A Appl. Sci. Manuf..

[32] Han Q.Q., Xu J.H., Shi J.Y., Zhou M., Wang H.B., Geng L., Xiong J.J., Du Z.L. (2025). Structural and hetero-interfacial engineering of magnetic bimetallic composites based polyurethane microwave absorbing coating for marine environment. Compos. Part A Appl. Sci. Manuf..

[33] Tahalyani J., Akhtar M.J., Kar K.K. (2022). Flexible, stretchable and lightweight polyurethane and graphene nanoplatelets nanocomposite for high performance EMI shielding application. Mater. Today Commun..

[34] Zhai Y., Zhu D.M., Chen Q., Nan H.Y. (2020). Efficiently enhanced microwave absorption of oriented flaky carbonyl iron&MoS_2_/polyurethane composite with thin thickness. Chem. Phys. Lett..

[35] Menon A.V., Madras G., Bose S. (2018). Shape memory polyurethane nanocomposites with porous architectures for enhanced microwave shielding. Chem. Eng. J..

[36] Gao Y., Wang C.Z., Li J., Guo S.Y. (2019). Adjustment of dielectric permittivity and loss of graphene/thermoplastic polyurethane flexible foam: Towards high microwave absorbing performance. Compos. Part A Appl. Sci. Manuf..

[37] Zheng J.J., Wei X.L., Li Y.C., Dong W.P., Li X.P., E S., Wu Z.Y., Wen J.M. (2021). Stretchable polyurethane composite foam triboelectric nanogenerator with tunable microwave absorption properties at elevated temperature. Nano Energy.

[38] Zhu G., Li H., Peng M., Zhao G., Chen J., Zhu Y. (2022). Highly-stretchable porous thermoplastic polyurethane/carbon nanotubes composites as a multimodal sensor. Carbon.

[39] Pan B., Lee K.J. (2023). Preparation of thermoplastic polyurethane blend foams with controlled hydrophobicity via vapor induced phase separation. J. Appl. Polym. Sci..

[40] Li Y.F., Li X.Y., Li Q.F., Zhao Y.H., Wang J.W. (2022). Low-energy-consumption fabrication of porous TPU/graphene composites for high-performance microwave absorption and the influence of Fe_3_O_4_ incorporation. J. Alloys Compd..

[41] Guo T., Li C.G., Yang J.L., Wang P.F., Yue J.L., Huang X.Z., Wang J., Tang X.Z. (2020). Holey, anti-impact and resilient thermoplastic urethane/carbon nanotubes fabricated by a low-cost “vapor induced phase separation” strategy for the detection of human motions. Compos. Part A Appl. Sci. Manuf..

[42] Shin B., Mondal S., Lee M., Kim S., Huh Y.-I., Nah C. (2021). Flexible thermoplastic polyurethane-carbon nanotube composites for electromagnetic interference shielding and thermal management. Chem. Eng. J..

[43] Liu L.J., Zhang X., Li X.Y., Wang Q., Li H.T., Zhang Y.P., Li Y.F., Li Q.F., Zhao Y.H., Wang J.W. (2025). Study on effect of boric acid on the stability of 1,5-naphthalene diisocyanate-based prepolymer and properties of the elastomers therefrom. Mater. Today Chem..

[44] Liu W.F., Zhao Z.P., Sun L., Wang M.Z. (2010). Formation of polyethersulfone film with regular microporous structure by water vapor induced phase separation. Chin. J. Chem. Eng..

[45] Zhang J.Q., Fan Z., Li B., Ren D.X., Xu M.Z. (2024). Study on structure–function integrated polymer-based microwave-absorption composites. Polymers.

[46] Li Y.F., Li X.Y., Kang M.Q., Zhao Y.H., Li Q.F., Wang J.W. (2023). Fabrication of flexible waterborne polyurethane/Fe-doped residual carbon from coal hydrogasification semi-coke composites for high-performance microwave absorption. Compos. Part A Appl. Sci. Manuf..

[47] Wang Q.Y., Wu X.F., Huang J., Chen S.Y., Zhang Y., Dong C.J., Chen G., Wang L.H., Guan H.T. (2022). Enhanced microwave absorption of biomass carbon/nickel/polypyrrole (C/Ni/PPy) ternary composites through the synergistic effects. J. Alloys Compd..

[48] Bi S., Zhao Y.K., Hou G.L., Zhang J.Y., Li H., Song Y.Z., Hou Z.L., Liu Z.H. (2022). Microwave absorption and mechanical properties of CNTs/PU composites with honeycomb structure. Appl. Compos. Mater..

[49] Grishchenko L.M., Diyuk V.E., Trachevskiy V.V., Zhytnyk D.O., Moiseienko V.A., Mischanchuk O.V., Skryshevsky V.A., Zaderko A.N., Boldyrieva O.Y., Lisnyak V.V. (2024). Polyurethane-based thin-film composites with carbon micro- to nanoscale fillers and their microwave properties. Mol. Cryst. Liq. Cryst..

[50] Ye X., Lv Y., Zhang L., Chen H., Chen S., Wu Y., Ma L.A., Wang Q. (2024). Hierarchical carbon nanotubes-modified heterogeneous composites derived from melamine-mixed ZIF-67/MXene for broadband microwave absorption. Carbon.

[51] Liu Z., Bai G., Huang Y., Li F., Ma Y., Guo T., He X., Lin X., Gao H., Chen Y. (2007). Microwave absorption of single-walled carbon nanotubes/soluble cross-linked polyurethane composites. J. Phys. Chem. C.

[52] Hussein M.I., Jehangir S.S., Rajmohan I.J., Haik Y., Abdulrehman T., Clément Q., Vukadinovic N. (2020). Microwave Absorbing properties of metal functionalized-CNT-polymer composite for stealth applications. Sci. Rep..

[53] Bhattacharya P., Sahoo S., Das C.K. (2013). Microwave absorption behaviour of MWCNT based nanocomposites in X-band region. Express Polym. Lett..

[54] Han Y.X., Guo H., Qiu H., Hu J.W., He M.K., Shi X.T., Zhang Y.L., Kong J., Gu J.W. (2025). Multimechanism decoupling for low-frequency microwave absorption hierarchical Fe-doped Co magnetic microchains. Adv. Funct. Mater..

