# Supplementary material for: Fabrication of 3D Porous and Flexible Thermoplastic Polyurethane/Carbon Nanotube Composites Towards High-Performance Microwave Absorption
Source: Molecules. 2025 Sep 3;30(17):3610. doi: 10.3390/molecules30173610 (PMC12430016; doi:10.3390/molecules30173610)
Supplement: Supplementary file 1 [file molecules-30-03610-s001.zip › molecules-3812138-supplementary.pdf]

Article

Fabrication of 3D Porous and Flexible Thermoplastic Polyurethane/Carbon Nanotube Composites Towards High-Performance Microwave Absorption

Yanfang Li [1,*], Yandong Xu [1], Guangming Wen [1] and Junwei Wang [2,*]

[1] Department of Chemical and Material Engineering, Lyuliang University, Lyuliang 033001, China
[2] State Key Laboratory of Coal Conversion, Institute of Coal Chemistry, Chinese Academy of Sciences, Taiyuan 030001, China
* Correspondence: liyanfang@llu.edu.cn (Y.L.); wangjw@sxicc.ac.cn (J.W.); Tel.: +86-351-4069680 (J.W.)

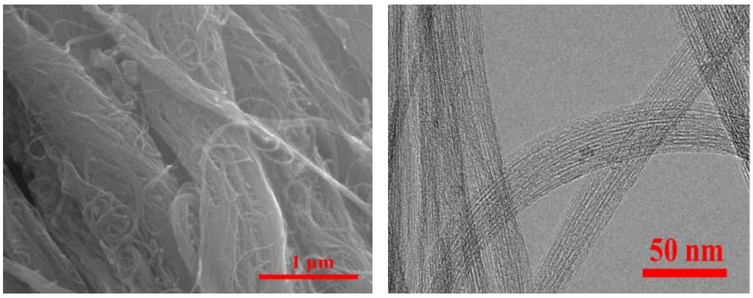

Figure S1. The SEM (a) and TEM (b) micrographs of CNT.

Table S1. Hydrogen bonging index and relative areas of the carbonyl stretching region in FT-IR spectra.

Samples	Fraction of peak area (%)		
	hydrogen-bonded carbonyl group	free carbonyl group	HBI
TPU	39.48	60.52	0.652
TPU/CNT-1	40.30	59.70	0.675
TPU/CNT-3	42.43	57.57	0.737
TPU/CNT-5	47.26	52.74	0.896

Table S2. Thermal characteristics of TPU and TPU-based composites from TG results.

Samples	$T_{5\%}$ (°C)	$T_{10\%}$ (°C)	$T_{50\%}$ (°C)	T_{max} (°C)
TPU	349.8	356.1	412.4	384.6, 428.8
TPU/CNT-1	330.6	338.9	399.8	418.3
TPU/CNT-3	312.8	323.2	384.1	393.7
TPU/CNT-5	288.1	303.4	380.9	382.4

 https://doi.org/10.3390/molecules30173610
